# Tumor infiltrating lymphocytes and radiological picture of the tumor

**DOI:** 10.1007/s12032-023-02036-3

**Published:** 2023-05-13

**Authors:** Karolina Frankowska, Michał Zarobkiewicz, Izabela Dąbrowska, Agnieszka Bojarska-Junak

**Affiliations:** 1 Department of Clinical Immunology, Medical University of Lublin, Lublin, Poland; 2 Department of Interventional Radiology and Neuroradiology, Medical University of Lublin, Lublin, Poland

**Keywords:** Tumor-Infiltrating lymphocytes, Tumor Microenvironment, Breast Neoplasms, Lung Neoplasms

## Abstract

Tumor microenvironment (TME) is a complex entity that includes besides the tumor cells also a whole range of immune cells. Among various populations of immune cells infiltrating the tumor, tumor infiltrating lymphocytes (TILs) are a population of lymphocytes characterized by high reactivity against the tumor component. As, TILs play a key role in mediating responses to several types of therapy and significantly improve patient outcomes in some cancer types including for instance breast cancer and lung cancer, their assessment has become a good predictive tool in the evaluation of potential treatment efficacy. Currently, the evaluation of the density of TILs infiltration is performed by histopathological. However, recent studies have shed light on potential utility of several imaging methods, including ultrasonography, magnetic resonance imaging (MRI), positron emission tomography-computed tomography (PET-CT), and radiomics, in the assessment of TILs levels. The greatest attention concerning the utility of radiology methods is directed to breast and lung cancers, nevertheless imaging methods of TILs are constantly being developed also for other malignancies. Here, we focus on reviewing the radiological methods used to assess the level of TILs in different cancer types and on the extraction of the most favorable radiological features assessed by each method.

## Tumor microenvironment—a complex entity

Tumor development takes place within what is known as the tumor microenvironment (TME). This abundant cellular environment, apart from the tumor cells, consists of other cellular and extracellular components. Among such cellular composition, the cluster of immune cells may be further distinguished including the tumor-associated macrophages (TAMs), lymphocytes B and T, as well as natural killer cells (NK cells) and natural killer T cells (NKT cells). While it is known that TAMs are responsible for processes essential for tumor invasion and the NK cells have a potential to kill cancer cells, the role of other cells is still under investigation [1, 2]. Between all those elements exists a complex net of interdependencies and those interactions are mediated by metabolites and cytokines generated by both tumor and TME cells [3]. Both the distribution of the immune infiltrate within the tumor and its composition are heterogeneous and differ between various tumors [4]. Moreover, the differences in the compositions of lymphocytes infiltrating the tumor within different subtypes of one type of cancer are observed [5].

**Fig. 1:**
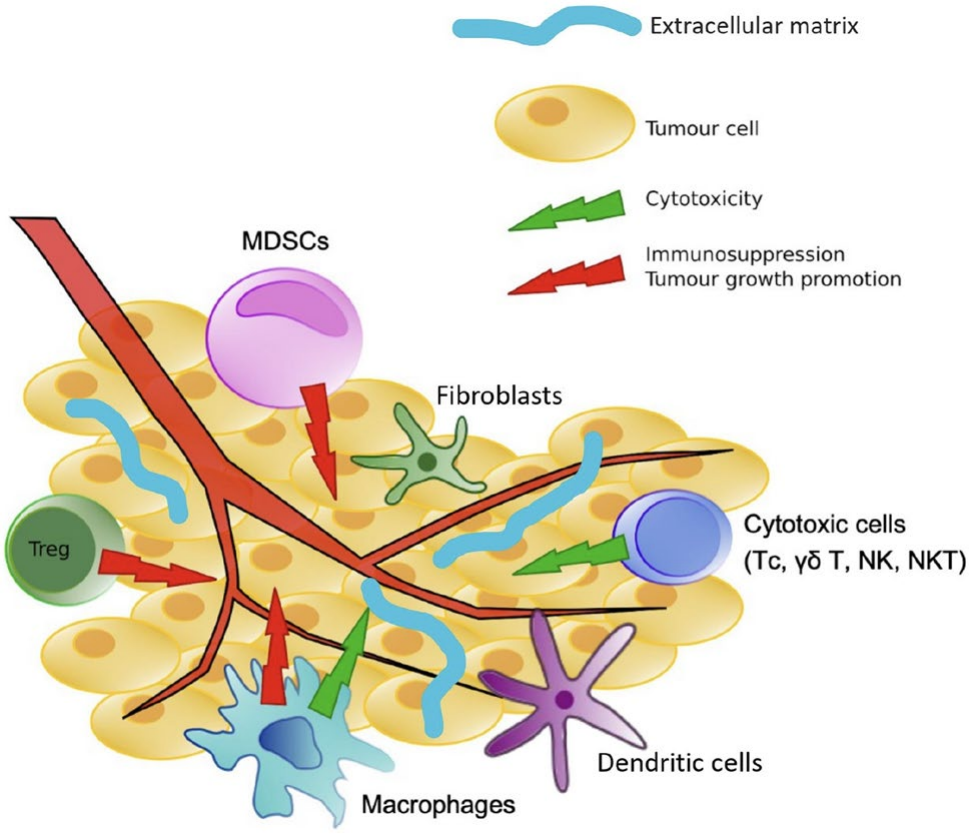
Tumor microenvironment. Tumor tissue hosts multiple different cell types—cancer cells, cytotoxic and regulatory lymphocytes, macrophages, myeloid-derived suppressor cells (MDSCs). A complex net of interactions exists in the tumor microenvironment—some cell types, e.g., macrophages can both exert anti-tumor effects (M1 macrophages) and promote tumor growth (M2 macrophages)

The significant part of immune cells within TME are tumor infiltrating lymphocytes (TILs), predominantly consisting of T, B, and NK cells (Fig. 1) [6, 7]. Depending on the subtype of these cells, they may present different activity patterns, as it is in case of individual subgroups of T lymphocytes which show different activity against tumor cells, thereby affecting patient prognosis [4]. High infiltration of CD8 + cytotoxic T cells is considered as a positive prognostic marker in various cancer types including breast cancer, ovarian cancer or colorectal cancer [8–10] as the role of these cells is associated with tumor destruction [11]. On the other hand, CD4 + regulatory T cells (Tregs) are responsible for promoting tumor progression, therefore their presence is a predictor of a worse prognosis for patients [12]. Although these trends are seen in many cancer types, the predictive properties of one type of immune cells are not always homogenous and for instance infiltration of CD8 + cells in renal cell carcinoma is a sign of unfavorable prognosis [13, 14].

The interactions between tumor cells and TME occur since the very early stages of tumor formation [15]. The existence of these phenomena is crucial from the clinical point of view for the assessment of cancer outcome and risk of progression, which indirectly depend on the nature of TME and TILs, as well as molecules released by and expressed on tumor cells [16]. The presence of TILs in in the tumor reflects the efficacy of the use of anti-cancer therapies including immune checkpoint inhibitors (ICIs) [17] and neoadjuvant chemotherapy (NAC) [18]. Additionally, TILs are the basis of the therapy of solid tumors, which uses the patients’ previously collected, multiplied, and properly stimulated TILs [19].

To make the above-mentioned therapies and their efficacy predictions as effective as possible it is important to accurately assess the lymphocytic infiltration of the lesion [20]. Current recommendations of the International TILs Working Group refer to the histopathological evaluation of TILs which is associated with biopsy or resection of the tumor [21, 22]. However, such an approach has some limitations in daily clinical practice due to the fact that the distribution of TILs within the tumor tissue is not homogenous and the biopsy material may not reflect the real infiltration of the tumor by TILs. Thus, core needle biopsy most often used in routine breast’s tissue evaluation is not a guarantee of a reliable result [23, 24].

Until now, multiple attempts have been made to use imaging methods in the assessment of TILs infiltration. Although the available studies focus most on breast and lung cancers, in which the widest range of imaging methods have been applied, other malignancies are also successfully included in such analysis. While non-invasiveness and accessibility of radiographic techniques support the advantage of such a solution, the question remains as to the effectiveness of imaging prediction of TILs density in different tumors, and thus the possibility of extracting specific features of radiological images that would indicate the TILs status of the tumor.

**Fig. 2:**
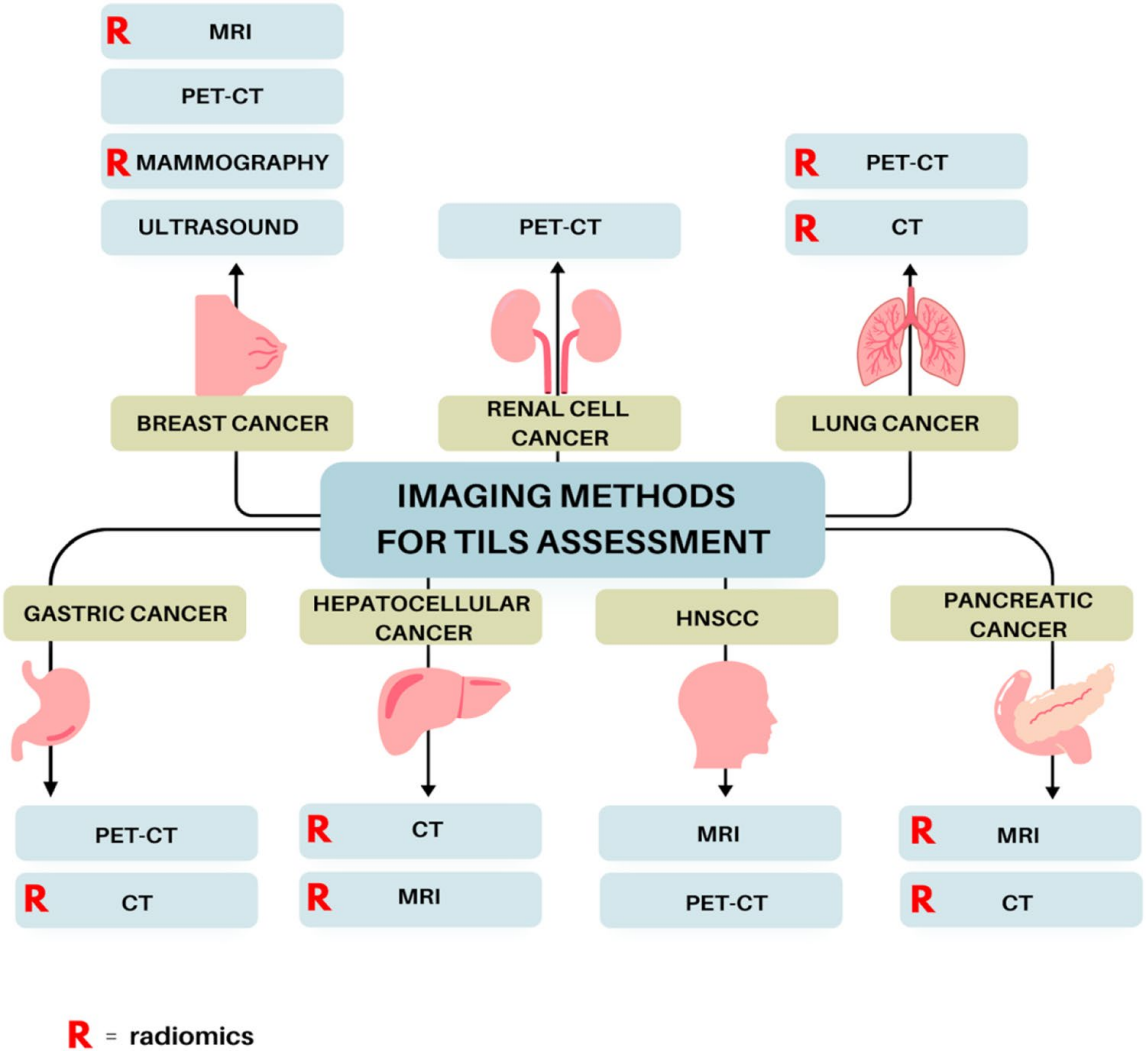
Graphic presentation of imaging methods used for TILs assessment

The aim of this review is to evaluate the usefulness of radiological imaging methods in accurately assessing the presence of TILs in the tumor (Fig. 2). It is expected that consistent features obtained by radiological imaging methods will allow us to use them in everyday oncological diagnostics.

## Breast cancer

Breast cancer is the most common cancer occurring in females. It accounts for about ¼ of all cancers occurring among women and it is also the leading cause of cancer deaths among women in most countries worldwide [25]. Based on its characteristics, breast cancer can be divided into a number of subtypes: luminal A, luminal B, human epidermal growth factor receptor 2 (HER2)-positive, and triple-negative breast cancer (TNBC) [26]. The amount of TILs varies depending on the type of breast cancer, especially high levels of TILs can be found in TNBC, as well as in HER2-positive cases [5, 27]. On the other hand, the breast cancer tumors classification system may also be related to the lymphocytic infiltration of the lesions. Hence, those with high- and low-TILs levels are defined as lymphocyte-predominant breast cancers (LPBC) and non-lymphocyte-predominant breast cancers (non-LPBC), respectively. Nevertheless, the precise criteria of such qualification are still under discussion [28].

From a clinical point of view, the density of TILs infiltration is relevant in the evaluation of the efficacy of anticancer therapies. The analysis of more than 3,700 cases of breast cancer patients proved that regardless of histologic type, lesions characterized by high infiltration of TILs responded better to neoadjuvant chemotherapy [18]. Moreover, such a feature has a predictive value for adjuvant chemotherapy [29] and increases the chance of success of ICIs therapy [30].

Breast tumor cancer morphology may be assessed with good effectiveness as a part of the radiological examinations [31]. Nowadays, imaging studies let us extract the imaging features being the predictors of molecular subtypes of breast cancer [32–36]. Thus, more and more researchers are investigating, whether the usage of preoperative imaging methods, including ultrasound (US), magnetic resonance imaging (MRI), positron emission tomography-computed tomography (PET-CT), and mammography examinations can be used to evaluate the extent of the density of TILs infiltration and become a new gold standard.

### Ultrasound

Ultrasound is a useful tool in both the detection of breast cancer and the monitoring of patients suffering from the disease [37–39]. All studies evaluating the potential relationships between TILs density and radiographic features in the ultrasound examination have shown some correlations, but the data are rather inconclusive (Table 1).

**Table 1:** Ultrasound features of TILs-rich breast cancers

Publication	Tumor size	Tumor shape	Tumor margins	Echogenicity
Fukui et al. 2019 [39]	Greater	Lobulated	Lobulated	Enhanced posterior echoes; weaker internal echo level
Fukui et al. 2021 [40]	–	Lobulated	Lobulated	Enhanced posterior echoes; weaker internal echo level
Çelebi et al. 2020 [43]	Greater	Oval/round	Circumscribed	Heterogeneous echogenicity
Jia et al. 2022 [44]	statistically non-significant difference	Oval/round	Circumscribed	Enhanced posterior echoes
Candelaria et al. 2022 [45]	–	Oval/round	Circumscribed or microlobulated	Enhanced posterior echoes; complex cystic and solid echo patterns

“-”—means unanalyzed feature

The initial study concerning the associations between breast cancer ultrasound features and TILs infiltration of the lesions was led by Fukui et al. in a group of women with different invasive breast cancer subtypes, predominantly classified as luminal ones. Their results indicated some differences between LPBC and non-LPBC lesions, like more lobulated shape including small lobulated areas (63,5% of LPBC cases vs. 10,8% of non-LPBC cases), lower internal echo level (40,4% of LPBC cases vs. 10,1% of non-LPBC cases), and more accentuated posterior echoes (82,7% of LPBC cases and 25,9% of non-LPBC cases). Moreover, tumors with higher TILs infiltration tended to have greater size (*p* = 0.01) [40].

Identical findings concerning the ultrasound features were obtained in a subsequent study conducted by the same authors with results enriched by contrast-enhanced ultrasonography (CEUS). The value of ascending slope (AS), defined also as a ratio of peak intensity of lesions’ brightness to time required to its reach, exceeding 10% was noted as significant and therefore set as a cutoff point [41]. Additionally, in the course of research, the authors proposed two similar scales to assess predisposition to a high density of TILs. In the first one, the highest scores are assigned to lesions showing very low internal echo level, highly accentuated posterior echoes and small lobulated shape, which qualify them for the group with the highest probability of rich TILs infiltration [40]. The second one, besides the above-mentioned features, also included AS values, and the lesions were scored if they were characterized with at least 10 percent of the parameter value [41]. So far, the utility of the first model scoring system was evaluated twice [42, 43]. The juxtaposition of proposed TILs-US score and conventional preoperative tissue biopsy provided comparable accuracy of both methods in predicting lesions rich in TILs (0.85 vs. 0.87 respectively) [42]. Moreover, the TILs-US score demonstrated great ability to reflect a pathological complete response to NAC in TNBC and HER2-positive cancer patients [43]. Nevertheless, although the scale seems to be a promising diagnostic tool, both proposed scales still require independent validation on bigger cohorts.

Based on the results from another study, it is suggested that tumor diameter higher than 2 cm in the ultrasound predisposes to higher levels of TILs. Nevertheless, in contrast to Fukui et al., the authors observed a relationship between high density of TILs and heterogeneous echogenicity of the lesion. Additionally, TILs-rich lesions were frequently characterized with round shapes and circumscribed margins [44].

Further, Jia et al. have focused on the differentiation between high- and low-TILs lesions in 267 breast cancer patients by using ultrasound and CEUS. They observed no differences in the size of the tumors between high- and low-TILs groups, and more common circumscribed margins, oval or round shapes and enhanced posterior echoes in the high-TILs group compared to low-TILs one. Moreover, the utilization of CEUS further developed the list of high TILs tumor characteristics—more regular shape, higher homogenous enhancement, clear margin after enhancement, as well as higher intensity peak [45].

Another recently published randomized clinical trial by Candelaria et al. performed on a group of 284 patients with TNBC focused on the association between ultrasound features being a part of the Breast Imaging Reporting and Data System (BI-RADS) scale and TILs infiltration. Oval or round shape, as well as circumscribed or microlobulated margins were a predictor of high lymphocytic infiltration. Additionally, tumors characterized by complex cystic and solid echo patterns, as well as enhancement posterior echoes more often were classified as rich in TILs [46].

The differences in characteristics of TILs-rich tumors can possibly be attributed to some important differences in study setups used by each group. Firstly, it is important to note that the authors have adopted different cutoff points for lymphocyte-rich tumors—on the level of 10% [44, 45], 20% [46] or 50% [40, 41]. Additionally, an overwhelming majority of study groups [40, 41, 44, 45] included all breast cancer subtypes and their percentage distributions in high-TILs groups differed significantly. Thus, the most accurate study model seems to be presented by Candelaria et al. as they decided to focus only on TNBC cases. It improves study comprehensibility and provides an opportunity for juxtaposing high-TILs features with typical ultrasound features reported for TNBC [47–49].

### Magnetic resonance imaging (MRI)

MRI is a useful tool in breast cancer imaging [50], especially in assessing the efficacy of NAC [51, 52]. Considering the strong relationship between the density of TILs infiltration and the response to NAC, the question arises, whether such a special role of MRI in evaluating the effectiveness of NAC is indirectly related to TILs.

Ku et al. conducted a study which found important relationships between morphological features of breast cancer in MRI and TILs levels. The data obtained from the patients with TNBC provided the following observations about the high-TILs cases: more oval or round margins in 23/50 patients (46%), absence of multifocality in 44/50 patients (88%), more circumscribed margins in 38/50 (76%), and homogenous internal enhancement 16/50 (32%) [53]. Those results raise new questions—whether such MRI features are indicators of high-TILs levels or are rather characteristic for this cancer subtype [35, 54]—especially that not all presented observations were fully in line with other studies. Çelebi et al. agreed only on more circumscribed margins and homogenous enhancement patterns in breast cancers rich in TILs [44]. In contrast, Choi et al. did not consider the utility of morphological characteristics on the tumors, highlighting the exclusive usefulness of assessment of background parenchymal enhancement (BPE) and peak enhancement [55].

Although current literature suggests no specific role of apparent diffusion coefficient (ADC) in distinguishing different breast cancer subtypes [56] and in predicting the response to NAC [57], this parameter distinguishing tissue, based on different diffusion of water molecules is widely considered in the context of TILs density evaluation. Çelebi et al. found ADC as the most valuable TILs density predictor and described an accuracy of 83% [44]. Similar results were reported by Fogante et al. [58]. Both studies were based on manually outlined 2 dimensional (2D) specifically regions of interest (ROI) to avoid areas of healthy tissue and hemorrhagic or necrotic parts of the tumor. They achieved specificity at the level of 73% and 80,3%, and sensitivity at the level of 60% and 67,8% respectively [44, 58]. The modified study regimen was presented by Tang et al. who proposed the use of the three-dimensional (3D) ROI with the following building of whole-lesion ADC histogram and analysis of parameters, including: 10th percentile ADC; mean ADC; 50th percentile (median) ADC; 90th percentile ADC; skewness and kurtosis. The authors created separate high-TILs indicators for luminal- and non-luminal cancer subtypes: higher 10th percentile ADC values and higher kurtosis values, respectively [59]. Taking into consideration described studies, ADC seems to be a promising method allowing the distinguishing of breast cancer tumors with different TILs infiltration. However, there are also studies which did not notice any correlation between high ADC values and high-TILs infiltration [53, 60, 61] but the background of these discrepancies is difficult to explain.

The potential utility of MRI with the assistance of computer-aided detection and diagnosis (CAD) programs has been described by Ku et al. Such technique allowed for an accurate assessment of not only morphological characteristics of breast cancer tumors but also kinetic features. Breast cancer tumors with high-TILs infiltration tended to present a washout enhancement pattern, while cases with low-TILs infiltration were more often characterized by persistent enhancement patterns. These differences were explained by the different vascularization of the tumors [62]. When analyzing the most valuable methods of TILs concentrations assessing, it is impossible not to mention the undoubted role of MRI-based radiomics models.

In their study, Bian et al. focused on the potential utility of a combination of clinical data and radiomics imaging features to different TILs levels differentiation. Two clinical factors: the size of the tumor and enhancement pattern, combined with 8 radiomics features served to build a nomogram model. It was characterized with the highest area under the curve (AUC) value (AUC = 0.84) compared to both clinical model (AUC = 0.72) and radiomics signature (AUC = 0.83) alone [63]. Xu et al. in their retrospective study on 172 breast cancer patients have drawn comparable conclusions. However, in this case, tumor diameter and estrogen receptor status were considered relevant clinical data and thus, with 7 radiomics features were a part of the nomogram model [64].

Another interesting radiomics model, created by Tang et al., was focused on the assessment of TILs level with the usage of different MRI sequences including diffusion-weighted imaging (DWI), T2-weighted imaging (T2WI), and dynamic contrast- enhanced (DCE)—also further subdivided into DCE_P1, pre-contrast; DCE_P2, super-early phase; DCE_P3, early phase; and DCE_P4-P6, delayed phase. The results obtained in single delayed phases (DCE_P4, DCE_P5, and DCE_P6) were characterized by the greatest effectiveness in predicting TILs levels. Finally, the analysis included 25 from among 6250 initial radiology features. Contrary to previous studies, considering the role of clinical factors (Ki-67 values and estrogen receptor values) did not improve the efficacy of predicting models [65].

The potential network between the MRI-based radiomics features and TILs concentration was also recently investigated in the group of TNBC patients who underwent preoperatively DCE-MRI. The Radiomics TILs score (Rad-TILs score), based on selected 3 radiomics features, was then juxtaposed with transcriptomics data concerning expression of several genes related to immunity. It has been shown that implemented radiomics model could be used to reflect lesions characterized with different immune patterns [66]. However, the role of MRI in estimating TILs density requires further analysis, particularly in the light of another recent report, which has shown that the combination of MRI radiomics and pathological TILs evaluation yielded the best results in assessing the efficacy of NAC compared to these methods alone. Thus, it can be concluded that the inclusion of pathological TILs evaluation in such a prediction process is still a guarantee of its substantial improvement [67].

### Positron emission tomography-computed tomography (PET-CT)

Immune cells and tumor cells compete for glucose, thus assessment of metabolic parameters in [^18^F]Fluorodeoxyglucose (^18^F-FDG) PET-CT may also give information about TILs [68, 69]. The initial study on the use of the PET-CT technique to visualize immune cells populations in breast cancer lesions drew only limited conclusions in this area. Namely, high maximum standardized uptake (SUVmax) values were connected with higher platelet to lymphocyte ratio (PLR) [70]. PLR and TILs seem to be closely associated in TNBC patients [71]. In fact, almost all further studies assessing the role of PET-CT in TILs level predicting, used the SUVmax assessment in their analysis and observed positive correlation between SUVmax values and TILs concentrations.

Murakami et al. observed higher ^18^F-FDG uptake with increasing TILs levels, which was reflected in higher SUVmax values [72]. With both whole-body PET-CT (WBPET) and dedicated breast PET-CT (DbPET) methods, it is possible to analyze the level of TILs by measuring the SUVmax—however the presence of a rich lymphocytic infiltrate has predisposed to higher SUVmax values only in DbPET [73]. Higher SUVmax values also correlated with higher TILs levels in study led by Park et al. Additionally, they have found that the increase in SUVmax value of 1 unit correlated with 14% higher probability of higher TILs [74]. Hirakata et al. also observed that high-TILs levels were strongly correlated with high SUVmax and that this parameter was in turn a predictor of more numerous CD8 + T cells among TILs [75].

Based on some likely existing dependencies between SUVmax values, some tumor characteristics (tumor size and Ki-67 labeling index) and TILs level, Sasada et al. created a scale named PET-TIL score. Their aim was to predict the effect of NAC. Although they reported that this scale was successfully used in evaluating the efficacy of NAC, it is difficult to establish whether their results were directly based on association with TILs, as TILs were not assessed directly in tissue slices [76]. Another PET-CT parameter which can be used as a predictor of TILs in women with breast cancer is spleen-to-liver (SLR) SUVmax ratios. Higher SLR values were more commonly observed in patients with low stromal TILs in comparison to patients with high stromal TILs [77]. High SLR in low-TILs patients may be explained by the abundance of immunosuppressive cells, e.g., myeloid-derived suppressor cells (MDSCs) [78]. Nevertheless, there are still some important discrepancies. Kajáry et al. noted no correlation between dynamic parameters measured in PET-CT and TILs. There is no clear explanation for this, but it is worth noting that this study was characterized by the smallest study group [79].

The common ^18^F-FDG PET-CT seems to offer some interesting and promising possibilities for quick and reliable assessment of TILs, but still requires significant optimizations with careful correlations to direct TILs measurements. Moreover, direct immuno-tracers should be considered as a possibly much better option [80].

### Mammography

There is also a considerable interest in the relationship between the different TILs densities in TNBC and mammographic radiomics features. Yu et al. found out that mammographic radiomics features such as uniformity, variance, gray-level co-occurrence matrix (GLCM) correlation, gray-level difference matrix (GLDM) low gray level emphasis, neighborhood gray-tone difference matrix (NGTDM) contrast and GLCM autocorrelation can be useful in distinguishing between high- and low-TILs levels [81]. Uniformity, mean and range GLDM low gray level emphasis of the craniocaudal (CC) view tended to be higher in the group where the level of TILs was lower. Contrastingly, among other significant variables, variance of the CC view, GLCM correlation of the mediolateral oblique (MLO) view and GLCM autocorrelation of the CC view showed higher values at higher lymphocyte infiltration density [81].

In the latest study, Yu et al. conducted a similar analysis exploring whether mammographic images can be used in the evaluation of the TILs status and investigating associations between mammographic radiomics features and TILs levels [82]. Similarly to previous findings [81] they confirmed that higher values of wavelet GLDM low gray level emphasis of the MLO view are associated with low levels of TILs [82]. In addition, they showed that gray-level run-length matrix (GLRLM) short run low gray level emphasis in the CC view, local binary pattern 2-dimensional (LBP2D) GLRLM short run high gray level emphasis of the CC view and LBP2D GLDM dependence entropy of the MLO view were more common in the low-TILs group. On the other hand, the wavelet interquartile range of the MLO view and LBP2D median of the MLO view tended to be higher in the high-TILs group [82].

Despite the promising results of these studies, they have important limitations, namely, they were single-centered retrospective studies and had small sample sizes [81, 82]. Moreover, there is an issue of radiomics classifiers calculation and consistently increasing risk of selection bias due to the using single largest slice in the mammographic images in that process [82].

## Lung cancer

Lung cancer is now a major worldwide health problem, and it is one of the leading causes of death [25]. The histological classification of lung cancer is based on a division into two main categories: non-small cell lung carcinomas (NSCLC, about 80 percent of all lung cancers) and small cell lung carcinomas (SCLC). NSCLC can be further divided into adenocarcinoma, squamous cell carcinoma and large cell carcinoma [83]. In recent years ICIs have been approved to treat lung cancer [84]. Therefore, there is a need to establish immune features useful for determining the prognosis of such therapy [85].

### Positron emission tomography-computed tomography (PET-CT)

^18^F-FDG PET-CT is widely used during the diagnosis process of lung cancer patients, thus it seems to be a good candidate to assess the status of the tumor microenvironment [86].

In a study conducted on the group of 55 NSCLC patients, both SUVmax and mean standardized uptake (SUVmean) tended to present higher values in tumors rich in CD8 + TILs and PD-1 + TILs [87]. Similar conclusions were reached by Wang et al. who in a larger group of 122 NSCLC patients found a positive relationship between CD8 + TILs density and SUVmax. They took into consideration the evaluation of metabolic tumor volume (MTV) and tumor lesion glycolysis (TLG) in tumors with different TILs levels and noted positive correlation between MTV and CD3 + TILs, as well as TLG and PD1 + TILs [88]. This trend regarding increased PET-CT features in high-TILs tumors was further developed in a study led by Zhou et al., in which 91 patients with NSCLC underwent the Dual-Time-Point FDG PET-CT (DTP FDG PET-CT). By this method the authors exploited the existence of parameters determined in early and delayed scans, as well as the changes between these two phases. The researchers’ aim was to evaluate the potential link between these parameters and different TME patterns. Along with TME observations, numerous positive associations between PET-CT parameters and different TILs populations have been found, e.g., CD8 + TILs and phase difference in SUVmean, CD8 + TILs and delayed phase SUVmean, PD1-TILs and early SUVmax, PD1-TILs and early SUVmean, PD1-TILs and phase difference in SUVmax, PD1-TILs and phase difference in SUVmean [89].

Also Castello et al. in their study decided to focus on unconventional PET-CT parameters and observed that in tumors characterized with high-TILs infiltration, the entropy tended to present higher values [90]. Nevertheless, there is no shortage of reports suggesting the lack of the usefulness of PET-CT in estimating the TILs infiltration. When analyzing the possible factors responsible for such results, there is a need to draw attention to the relevance of the type of tracer used in the PET-CT examinations. Shimizu et al. performed a PET-CT examination using 3-[^18^F]Fluoro-α-methyl-L-tyrosine ([^18^F]FAMT) and no significant correlations between TILs and SUVmax in [^18^F]FAMT PET-CT were observed [91]. Moreover, the selection of lung cancer subtypes also seems to affect the final outcomes concerning the TILs imaging. Kasahara et al. reported the following association in SCLC patients—the higher density of TILs corresponded to the lower ^18^F-FDG uptake [92]. Similarly, Kasahara et al. in their other study [93] and Kaira et al. [94] limited their studies to only one lung cancer subtype: pulmonary squamous cell carcinoma and pulmonary adenocarcinoma, respectively. Thus, we hypothesized that it was the reason for the missing link between ^18^FDG-PET features and TILs density [93, 94].

Interestingly, a recently published article reported that PET-CT can be used not only to classify tumors on the basis of immune cell density but also to classify them by the presence of products secreted by these cells, as shown in the study conducted by Dönez et al. They found that the presence of CCL-18 produced by tumor-associated macrophages (TAMs) in NSCLC is reflected in different features of ^18^FDG PET-CT images [95].

### Radiomics

Radiomics represent another widely discussed method in distinguishing between lung cancer tumors with low and high lymphocytic infiltration (Table 2). Zhou et al. designed a study, in which based on radiomics features extracted from PET-CT, differentiate the NSCLC tumors characterized by different TME patterns. They found 37 radiomics features that correlated with CD8 + TILs and in total 68 both PET- and CT-radiomics features that correlated with PD1-TILs. Moreover, the juxtaposing of the analyzed correlations, highlights the features for which the correlations with CD8 + TILs and PD1-TILs were the strongest—NGLDM_cotrast and peak standardized uptake value (SUVpeak), respectively [96].

**Table 2:** Radiomics in differentiation of NSCLC cases with different TILs levels

Publication	Diagnostic method	The number of patients	Main results
Khorrami et al. 2020 [96]	CT	139 patients with NSCLC (from among them, 36 cases were analyzed for the association between radiomics features and TILs infiltration)	They extracted 76 radiomics features and found that peritumoral Gabor feature (feature related to the tumor structure) was a strong predictor of TILs density infiltration
Mazzaschi et al. 2020 [97]	CT	100 patients with NSCLC	They found well defined margins, the presence of CT evidence of tumor effect, as well as radiomic features related to Non-uniformity to be predictors of rich immune cells infiltration
Chen et al. 2022 [98]	CT	117 patients with NSCLC	Heterogenous dependencies and complicated texture were related to tumors characterized with high CD8 + TILs infiltration
Yoon et al. 2020 [99]	CT	149 patients with NSCLC	239 CT-based radiomics features have been extracted. The significant relationships between TILs levels in tumors and imaging features were related only to Th2 cells, in contrast to Th1 and Tc cells

The distinguishing accuracy of radiomics was further focused on the CT-based radiomics models [97–100]. The analysis of the 36 NSCLC tumors specimens in the study by Khorrami et al. was used for extraction of the most essential Delta-radiomics (DelRADx) features. The authors observed high-TILs infiltration to be connected with Gabor feature which is related to the texture of the lesion [97].

In the study by Mazzaschi et al. the focus has been on the extraction of both the semantic CT features (CT-SFs) and radiomic CT features (CT-RFs) related to abundant TILs infiltration. The evaluation of the first features subgroup brought the following details concerning high-TILs density tumors imaging: well defined margins and the presence of CT evidence of tumor effect. Additionally, the authors observed that among CT-RFs, these related to NonUniformity were predictors of infiltration rich in CD8 + TILs [98].

Big potential related to the utility of radiomics in NSCLC patients TILs levels prediction was demonstrated in the study by Chen et al. in which 117 patients were qualified. Lesions with high CD + 8 TILs infiltration in CT-based radiomics have presented a more heterogeneous and complicated texture [99].

Interesting results related to the predictive role of radiomics in different subpopulations of immune cells in NSCLC cases were reported by Yoon et al. The authors compared the efficacy of the applied methods for Th1, Th2, and Tc cells infiltration prediction. In both the training and the validation cohorts the researchers proposed the use of both contrast and non-contrast CT. Finally, radiomics proved to be a usable method only in relation to Th2 cells level prediction [100].

## Other cancers

### Pancreatic cancer

Due to the highest incidence of pancreatic ductal adenocarcinoma (PDAC) among various types of pancreatic cancers, PDAC and pancreatic cancer are generally understood synonymously [101]. The cases of PDAC are not the leading cause of cancer-related deaths. Nonetheless, due to the fact that the number of PDAC related deaths is roughly equivalent to the number of diagnosed cases, it is a public health concern [25]. The implementation of standard treatment methods, including chemotherapy and surgical regimen, in PDAC patients yields unsatisfactory results [102, 103]. As the application of immunotherapy does not guarantee a success, there is a need to qualify those patients, who would benefit the most from specific therapies [104]. According to the recently published studies, TILs density in the tumor environment may represent such a predicting factor in PDAC patients [105].

All studies which investigated the role of imaging methods in TILs levels prediction in PDAC cases focused on the use of radiomics and artificial intelligence (AI) to analyze the radiomics [106–110] and the majority of them suggested the usage of radiomics models combined with non-radiomics features obtained from imaging studies.

In order to establish a predicting model of TILs levels, Bian et al. have built linear discriminant analysis (LDA) model based on 13 radiomics features (4 first-order statistical features, 4 GLCM features, 3 Gy-level size-zone matrix (GLSZM) features, and 2 GLRLM features) and mixed model including both the above-mentioned radiomics characteristics and 12 features obtained from non-contrast MRI examination. The comparative analysis of AUC in validation groups from both proposed models did not show differences in their efficacy (AUC = 0.76 vs. AUC = 0.69, respectively) [106]. Further improved effectiveness of radiomics implementation was shown in combined usage of 12 radiomics features and clinical features (location of the lesion and tumor size). Hence, the same authors reached AUC = 0.79 [107]. The similar satisfactory value of the radiomics method (AUC = 0.67) was obtained in the prediction model linking 10 CT-based radiomics features and tumor size [108]. The only research in which apart from 37 radiomics characteristics of computed-tomography, no other imaging-derived features were included in the analysis, was led by Bian et al. Although the authors found no other imaging features to be positively associated with TILs, further considering AUC values (0.79), such prediction model seems to be comparatively reliable [109]. Another interesting machine learning model was proposed by Li et al. via non-contrast MRI in 156 PDAC patients. The authors focused on CD20 + B Cells level reflection in radiological images created through radiomics development [110].

There were numerous attempts to prepare a good machine learning model that could predict TILs density in PDAC based on radiomics features and some basic clinical information, e.g., tumor size based on both MRI [106, 107, 110] and CT [108, 109] findings. Overall, each of the models provided satisfactory end-results. Nevertheless, those studies were based on small samples and most importantly, they lacked prospective controls, thus the findings should be treated with caution.

### Gastric cancer

Gastric cancer is the sixth most commonly diagnosed cancer worldwide with about twice as many new cases diagnosed in men as in women. In addition, gastric cancer is the third most common cause of death from all cancers [25]. Although levels of immune cell infiltration in gastric cancer are a useful prognostic tool, only two retrospective studies evaluating the potential utility of imaging features in TILs density prediction have been published so far [111, 112]. Lee et al. conducted a study on the group of 56 patients with gastric cancer and noted the positive association between SUVmax on ^18^F-FDG PET-CT and regulatory T cells in gastric cancer. In addition, there was also a positive correlation between high SUVmax values and the presence of CD3 + T lymphocytes but this association was negligible [111]. So far, the largest published study focusing at all on the usefulness of imaging methods in TILs levels prediction, was related to the development of relevant radiomics features of gastric cancer tumors. The authors proposed 13 radiomics characteristics derived from contrast-enhanced CT which led to radiomics immunoscore (RIS) establishment. The data of 1778 patients was analyzed and the obtained results indicated that gastric cancer patients demonstrating low RIS values were more likely to present low CD3 + , CD8 +, and CD45RO + lymphocytic infiltration [112]. Although the revealed observations were encouraging, it should be noted that only selected lymphocyte subpopulations were analyzed which does not equal the assessment of all TILs. Thus, interesting results of a recent meta-analysis suggest that although TILs are largely prognostic markers in gastric cancer, in contrast Tregs do not have a significant impact on patients’ survival [113].

### Head and neck squamous cell carcinoma

In the USA, head and neck squamous cell carcinoma (HNSCC) accounts for 3 percent of all diagnosed cancers. More than half a million new cases of HNSCC are diagnosed worldwide each year [114]. Since the presence of TILs is also a prognostic marker in HNSCC patients [115, 116], a study was conducted to evaluate the connection between the density of TILs infiltration and radiological features on MRI images. Indeed, there was an association between different T2WI-derived parameters and TILs within the tumor compartment, however no such correlation was demonstrated in T1-derived images. On the other hand, images in T1WI-derived sequences correlated with tumors with high expression of TILs within the stroma compartment (stroma-rich tumors). Additionally, it was observed that kurtosis tended to be higher in the tumors with high expression of TILs in the tumor compartment in T2WI-derived images and this association was not observed in stroma-rich tumors [117].

In addition to the assessment of the imaging features indicative for the presence of TILs by MRI, the analysis of TILs status in oral squamous cell carcinoma was also performed by PET-CT. Togo et al. found that tumors with low CD8 + TILs infiltration tended to have higher ^18^FDG uptake than lesions with high TILs [118].

### Hepatocellular cancer (HCC)

Liver cancer also ranks among the top ten most prevalent cancers worldwide [25]. Hepatocellular cancer (HCC) comprises about 85% of total liver cancer cases [119]. Currently, HCC management is applied to suit different HCC stages and other clinical data, especially immunotherapies should be carefully chosen [120, 121].

So far, previous studies suggested TILs to be helpful as a predictive parameter [122–124]. However, despite the above-mentioned encouraging results, only two studies focused on the relevance of the imaging-based assessment of TILs infiltration [125]. Liao et al. constructed a CT-based radiomics model based on the 7 extracted features. Such a developed RIS model showed a correlation with CD8 + TILs and the AUC values of the applied prediction model in the training cohort and validation cohort were 0.751 and 0.705, respectively [125].

Nonetheless, it seems that the final radiomics model proposed by Chen et al. was characterized with greater predictive value. The authors compared the efficacy of the following 3 different radiomics-based models: intratumoral radiomics one, combined radiomics one, and combined radiomics-based clinical one, to predict the immunoscore which reflected the TILs levels. The last one was found to be the most relevant, as the AUC value in the validation set reached 0.934, in comparison to the AUC = 0.640 and AUC = 0.899 in the intratumoral radiomics and combined radiomics, respectively [126].

### Renal cell cancer (RCC)

Among a variety of renal cell carcinoma (RCC) subtypes, clear cell Renal Cell Carcinoma (ccRCC) represents the vast majority of diagnosed RCC cases, accounting for about ¾ of all [127]. Although the development of ICIs therapy provided promising results in ccRCC patients, a small group of patients is benefiting from this type of therapy [128, 129]. Currently the efficacy of TILs density infiltration in predicting the effectiveness of ICIs therapy is a subject of an on-going discussion [130, 131]. Until now, to the best of authors’ knowledge only one study evaluating the usage of imaging methods in assessment of TILs density in ccRCC patients was published. Wu et al. led a study on the group of 90 patients with ccRCC with preoperatively performed ^18^F-FDG-PET-CT examination. They observed higher chances to detect lesions with high-TILs infiltration in patients with higher SUVmax values [132].

## Discussion

The wide range of imaging methods available in everyday clinical practice opens new prospects for their usefulness in assessment of TILs infiltration in different cancer types. However, the question remains, whether it is possible to identify methods or even specific parameters, which would be the most appropriate and could establish a standard in the assessment of TILs in different types of cancer.

When analyzing all the above-mentioned studies, it can be concluded that there is no single imaging method effective in visualizing TILs in all cancer types. Nevertheless, some tendencies regarding efficacy can be identified for individual tumor types. On the example of breast cancer, we found that studies conducted with the use of PET-CT imaging present highly consistent results, therefore various parameters regarding glucose metabolism seem to reflect the TILs status with great efficacy. Additionally, in breast cancer attention should be paid to methods based on radiomics or AI, which are highly individualized and allow the assessment of very detailed characteristics of the tumor. So far, MRI and mammography have been included in the development of models involving AI techniques. Therefore, one of the solutions could be to combine AI with other imaging methods in the future, to achieve their improved results.

Similar observations were received regarding radiological assessment of TILs in lung cancer. Although the spectrum of analyzed methods was less extensive, as included only PET-CT and AI-based techniques, the methods engaging AI seem to present satisfactory results. Additionally, especially important in the case of breast and lung cancers, due to their histological differentiation, is to conduct research on homogenous groups including single subtypes of these cancers.

What constitutes a restriction in indication of the most appropriate TILs imaging method in other analyzed cancer types including pancreatic cancer, gastric cancer, HNSCC, hepatocellular cancer or renal cell cancer is small number of studies, which additionally are often limited to one type of investigated technique.

In sum, the assessment of TILs with the use of imaging methods in general is a promising management. Nevertheless, we cannot indicate specific imaging methods and parameters that could be applied in radiological assessment of TILs in various tumors. Especially many hopes are connected to new techniques including AI or radiomics which can be seen in example of pancreatic cancer, where all research on this cancer was conducted based on such imaging methods. Although, we assume that histopathological examination will continue to be the gold standard, radiological evaluation should be still investigated in further studies led on a greater number of patients.

## Conclusion

Despite a significant number of available studies, it is impossible to indicate fully reliable imaging features to differentiate tumors with high-TILs level. Ultrasound, MRI or PET-CT imaging methods present rather inconsistent observations. Some hopes are connected with radiomics and artificial intelligence. Regardless of tested methods, all studies so far lacked the prospective group. Thus, future studies should include not only more significant patient groups, but also should be based on both retrospective and prospective data.

## Data Availability

Not applicable.
